# Lutembacher syndrome presenting as heart failure in a young Nigerian at Obafemi Awolowo University Teaching Hospitals Complex (OAUTHC), Ile-Ife: a case report

**DOI:** 10.11604/pamj.2022.41.342.29903

**Published:** 2022-04-28

**Authors:** Opeyemi Ezekiel Ojo, Anthony Olubunmi Akintomide, Rasaaq Ayodele Adebayo, Olumide Akinniyi Akinyele, Ikponmwosa Godfrey Akhionbare, Oyeronke Titilope Williams, Busayo Onafowoke Oguntola, Obafemi Sunday Adesanya, Adebiyi Lukman Obasanjo

**Affiliations:** 1 Cardiology Unit, Department of Medicine, Obafemi Awolowo University Teaching Hospitals Complex (OAUTHC), Ile-Ife, Osun State, Nigeria

**Keywords:** Lutembacher syndrome, rare case, heart failure, late presentation, case report

## Abstract

Lutembacher syndrome (LS) is a rare syndrome comprising a combination of atrial septal defect (ASD) and mitral stenosis. We present the case of a 28-year-old man, who presented with progressively worsening dyspnea of 2 months associated with orthopnea, paroxysmal nocturnal dyspnea, bilateral leg swelling and productive cough. Chest X-ray revealed plethoric lung fields with prominent pulmonary conus and cardiomegaly. Transthoracic echocardiography revealed a large ostium secundum ASD with left to right shunt, mild mitral stenosis, severe mitral and tricuspid regurgitations and pulmonary hypertension. A diagnosis of Lutembacher syndrome in heart failure with pulmonary hypertension was made. The patient was managed conservatively, but declined surgery primarily because of financial reasons. This rare case of LS presenting with heart failure and complicated by pulmonary hypertension is the first reported case in our centre and our region. The patient's inability to afford the cost of definitive care posed a significant problem in his management.

## Introduction

Lutembacher syndrome (LS) is a rare cardiac clinical condition characterised by any combination of atrial septal defect (ASD) (congenital or iatrogenic) and mitral stenosis (MS) (congenital or acquired) [1,2]. However the combination of a congenital ASD (usually secundum type) and acquired MS (usually rheumatic) is the common well recognized pattern [2]. Although LS might be common in some part of the world but it is not commonly seen in this environment probably due to limited appropriate equipment and inadequate skilled personnel to make the diagnosis when present. Generally, LS is a very rare clinical entity with very few case reports across the globe [2,3]. We present a case of a young adult male with this rare heart disease at Obafemi Awolowo University Teaching Hospitals complex (OAUTHC), ile-ife located in South Western region of Nigeria.

## Patient and observation

**Patient information:** a 28-year-old male who presented at the Adult Accident and Emergency Unit of Obafemi Awolowo University Teaching Hospital Complex (OAUTHC), Ile-Ife, Osun State, Nigeria, on account of progressively worsening dyspnea on exertion of 2 months associated with orthopnea, paroxysmal nocturnal dyspnea (PND) and bilateral leg swelling. He also had cough of 2 weeks productive of whitish and frothy sputum. There was no history of fever, facial swelling or reduction in urine output. No known prior history of recurrent sore throats or skin ulcers. He was not previously diagnosed to have hypertension or diabetes. He had history of significant alcohol intake for about 5 years (average of 120 g per day for 5 years), however no history of smoking.

**Figure 1:**
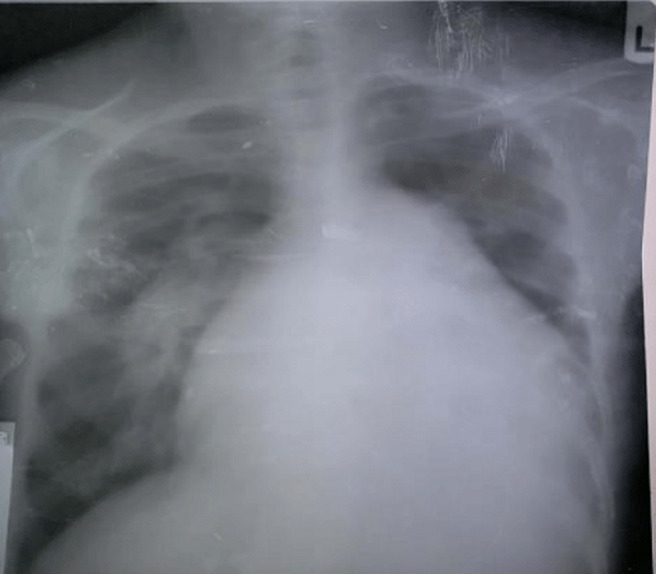
chest X-ray P/A view showing bulged pulmonary conus, cardiomegaly with CTR (cardiothoracic ratio) of 0.8 and pulmonary plethora

**Figure 2:**
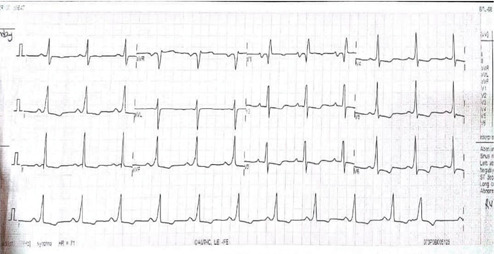
a twelve-lead ECG showing LA enlargement, right ventricular hypertrophy and incomplete right bundle branch block (QRS duration is 116ms)

**Figure 3:**
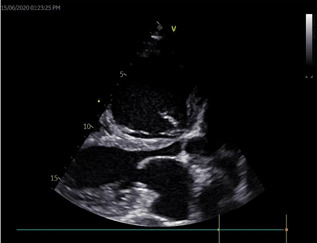
PLAX view 2D ECHO picture showing sclerosed MV leaflets and subvalvular structure with hockey stick appearance of AMV, restricted MV opening and dilated RV

**Figure 4:**
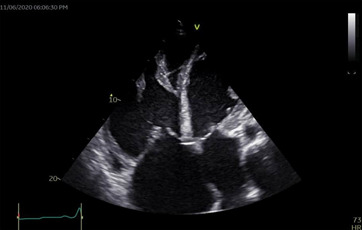
two-dimensional echocardiography (apical four-chamber view) showing large secundum ASD of 27mm, mild dilation of the left atrium, severe right ventricular and right atrial dilation

**Figure 5:**
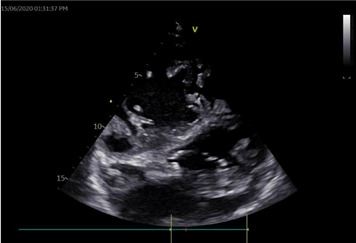
two-dimensional echocardiography (parasternal short axis view) showing mild mitral stenosis (MVA=3cm^2^ by planimetry) and bicommisural calcification of the MV

**Figure 6:**
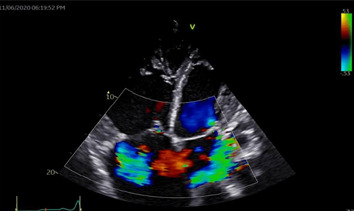
two-dimensional ECHO with colour doppler (apical four-chamber view) showing flow across the ASD and also severe mitral and tricuspid regurgitations

**Clinical findings:** on examination at admission, he was dyspneic and cyanosed with a respiratory rate of 30 breaths/min and SPO_2_ of 78% (room air). He had a normal volume regular pulse of 110 beats/min, blood pressure of 110/60mmHg (sitting) and bilateral pitting pedal edema up to the knees. On cardiac examination, Jugular venous pressure (JVP) was elevated to the angle of the jaw, apex beat was located at 7^th^ left intercostal space, anterior axillary line and heaving. There was left parasternal heave and palpable P2. Auscultation revealed a third heart sound with pansystolic murmur grade 3/6 in both mitral and tricuspid areas and a systolic ejection murmur in the pulmonary area. Other auscultatory findings include widely split and fixed S2, loud P2 and bibasal fine crackles. Abdominal examination showed tender soft pulsatile hepatomegaly and ascites. Other systemic examinations revealed no abnormal findings.

liver function test showed mild elevation of total and conjugated bilirubin, however other liver enzymes were normal. Other laboratory results including glucose, renal function, complete blood count, clotting profile, fasting lipid profile and serology (hepatitis B, C and HIV screening) tests were normal. Chest X-ray revealed plethoric lung fields with prominent pulmonary conus and cardiomegaly with a cardiothoracic ratio of 0.80 (Figure 1). A twelve-lead electrocardiogram (ECG) showed left atrial (LA) enlargement, right ventricular hypertrophy and incomplete right bundle branch block (Figure 2). Transthoracic echocardiography (ECHO) revealed a large ostium secundum ASD (size 27 mm) with left to right shunt, mild MS (mitral valve area by planimetry and pressure half-time are 3.0 cm^2^ and 2.4 cm^2^. Mean pressure gradient=0.74 cm^2^), severe mitral and tricuspid regurgitations, moderate pulmonary regurgitation and pulmonary hypertension (estimated mean pulmonary arterial pressure=51mmHg). Mitral valve (MV) leaflets and subvalvular structures were thickened and partly calcified, with uniformly restricted movements of the MV and bi-commissural calcification. Other cardiac valves were normal. Left ventricular systolic function was preserved with an ejection fraction of 67%, however, right ventricular systolic function was impaired (TAPSE= 15 mm). There was biventricular diastolic dysfunction. Dilated right ventricle, left and right atria and spontaneous echo contrast in all cardiac chambers were also noted. The ECHO image findings above are shown in Figure 3, Figure 4, Figure 5, Figure 6.

**Diagnosis:** an initial provisional diagnosis of congestive cardiac failure (NYHA IV) due to rheumatic valvular heart disease was initially made, with congenital heart disease (ASD) and alcoholic cardiomyopathy kept as a remote differential diagnosis. After the ECHO imaging was done, the final diagnosis of Lutembacher syndrome in heart failure with pulmonary hypertension was made.

**Therapeutic interventions:** antifailure drugs including ramipril, bisoprolol, spironolactone, furosemide, digoxin as well as warfarin and oxygen therapy were administered. After 6 days of admission, his clinical parameters were stable as he was no longer dyspneic at rest. Likewise, orthopnea, PND, leg swelling and cyanosis resolved, pulse rate and BP (sitting) were 80bpm and 110/70mmHg respectively and SPO_2_ in room air was 97%.

**Follow-up and outcome of interventions** was discharged in NYHA class II and was followed up both in cardiology and cardiothoracic surgery clinics. Patient was later scheduled for surgery by the cardiothoracic surgical unit, but he declined primarily for financial reasons.

**Patient perspective:** he adjudged to significant clinical improvement and improved exercise tolerance after pharmacological treatment. However, He declined surgery because he could not afford the cost of surgery due to his poor socioeconomic background and lack of health insurance to cover the cost of surgery.

**Informed consent:** a written informed consent was obtained from the patient for publication of his clinical details. Approval was obtained from the ethics and research committee of OAUTHC with ethics certificate no: IRB/IEC/0004553.

## Discussion

Pattern of prevalence of congenital heart disease (CHD) reported in Nigeria varies according to regions. However, most of the reports indicated that ventricular septal defect is the most common CHD while tetralogy of Fallot is the most common cyanotic CHD [4,5]. Although there is no report on the prevalence of LS in Nigeria or sub-Saharan Africa, it´s been predicted to be likely prevalent in this region due to high prevalence of rheumatic heart disease [1]. Lutembacher syndrome is a rare clinical entity, with a study in the United States putting the incidence at 0.001 per million population [6]. Rene Lutembacher in 1916 first published a case report describing the combination of congenital ASD (secundum type) and congenital MS in a 61-year-old woman who had been pregnant seven times [7]. However, over the years, a number of literature have described other forms of LS including acquired mitral stenosis (usually rheumatic in origin) which is the commonest form of MS seen in LS, acquired/iatrogenic ASD (usually secondary to trans-septal puncture during mitral valvuloplasty for acquired MS) and reverse LS (right to left shunt ASD and tricuspid stenosis) [3,8]

In low-income countries like Nigeria with relatively high burden of rheumatic heart disease, LS is most frequently first diagnosed in symptomatic young adults [3,9]. Patients may present with varied symptoms including fatigue, exercise intolerance or frank symptoms of heart failure. Our patient presented in his third decade of life with frank heart failure symptoms. Although past history of rheumatic fever could not be established from the clinical history which has been reported to be absent in 60% of patients [10], however, the age of the patient, increased prevalence of rheumatic heart disease in the region and echocardiographic findings of pathological severe mitral regurgitation, thickened anterior valve leaflets (>3 mm), chordae thickening and restricted leaflets motion makes us conclude the mitral stenosis was rheumatic in origin based on world heart federation criteria for diagnosing rheumatic heart disease [11]. Atrial septal defect is believed to be congenital in origin, as the patient had no previous history of trans-catheter intervention. Other characteristic features of pure MS such as diastolic rumbling murmur, loud first heart sound, markedly dilated LA, lung congestion and hemoptysis are usually less common or delayed due to decompression of the LA via the ASD which eventually results in markedly dilated right atria and ventricle and right heart failure [2].

Percutaneous transcatheter therapy has become the preferred non-surgical approach to managing the disease with fewer complications, provided there are no contraindications to either catheter closure of ASD or catheter balloon mitral valvuloplasty [12]. Transthoracic echocardiography done for the patient showed bicommisural calcification and significant mitral regurgitation, which contraindicated balloon mitral valvuloplasty. Kukarni *et al*. reported that LS presenting with heart failure and pulmonary hypertension are poor prognostic indices, as in our index patient [13]. Generally, it has been observed that quite a number of patients in Nigeria and West African subregion present late to the hospital due to a number of reasons including traditional/herbal therapy at the early stage of clinical symptoms and inability to afford orthodox medicine [3,14,15]. Our patient could not undergo definitive treatment (surgery) as planned by the cardiothoracic surgery unit due to inability to afford cost of care. It has been observed in developing country that inability to afford cost of care contribute to poor prognostic outcome. Alternative sources of funding/assistance to such a patient through financial support by the Government/Non-Government Organizations and collaborative partnerships with foreign donor agencies should be considered [14,16]. Despite the aforementioned factors which shorten the lifespan of patients with LS, there are case reports including the index reported case in Nigeria who presented for the first time in late adulthood [12,17].

## Conclusion

We have presented a case of Lutembacher syndrome in heart failure with pulmonary hypertension. The management of this patient is complicated by his late presentation and inability to pay for definitive care (surgery).
